# Proteomic Analysis of C2C12 Myoblast and Myotube Exosome-Like Vesicles: A New Paradigm for Myoblast-Myotube Cross Talk?

**DOI:** 10.1371/journal.pone.0084153

**Published:** 2014-01-02

**Authors:** Alexis Forterre, Audrey Jalabert, Emmanuelle Berger, Mathieu Baudet, Karim Chikh, Elisabeth Errazuriz, Joffrey De Larichaudy, Stéphanie Chanon, Michèle Weiss-Gayet, Anne-Marie Hesse, Michel Record, Alain Geloen, Etienne Lefai, Hubert Vidal, Yohann Couté, Sophie Rome

**Affiliations:** 1 CarMeN Laboratory (INSERM 1060, INRA 1362, INSA) University of Lyon, Faculté de Médecine Lyon-Sud, Oullins, France; 2 Centre Commun d’Imagerie de Laënnec (CeCIL), SFR Santé Lyon-Est, University of Lyon, Lyon, France; 3 CEA, IRTSV, Laboratoire Biologie à Grande Echelle, Grenoble, France; 4 INSERM-UMR 1037, Centre de Recherche en Cancerologie de Toulouse (CRCT), Toulouse, France; 5 INSERM, U1038, Grenoble, France; 6 Grenoble Alpes University, IRTSV, Laboratoire Biologie à Grande Echelle, Grenoble, France; 7 Centre de Génétique et de Physiologie Moléculaire et Cellulaire (CGPhiMC), CNRS UMR5534, University of Lyon, Lyon, France; Institut de Myologie, France

## Abstract

Exosomes are nanometer-sized microvesicles formed in multivesicular bodies (MVBs) during endosome maturation. Exosomes are released from cells into the microenvironment following fusion of MVBs with the plasma membrane. During the last decade, skeletal muscle-secreted proteins have been identified with important roles in intercellular communications. To investigate whether muscle-derived exosomes participate in this molecular dialog, we determined and compared the protein contents of the exosome-like vesicles (ELVs) released from C2C12 murine myoblasts during proliferation (ELV-MB), and after differentiation into myotubes (ELV-MT). Using a proteomic approach combined with electron microscopy, western-blot and bioinformatic analyses, we compared the protein repertoires within ELV-MB and ELV-MT. We found that these vesicles displayed the classical properties of exosomes isolated from other cell types containing components of the ESCRT machinery of the MVBs, as well as numerous tetraspanins. Specific muscle proteins were also identified confirming that ELV composition also reflects their muscle origin. Furthermore quantitative analysis revealed stage-preferred expression of 31 and 78 proteins in ELV-MB and ELV-MT respectively. We found that myotube-secreted ELVs, but not ELV-MB, reduced myoblast proliferation and induced differentiation, through, respectively, the down-regulation of Cyclin D1 and the up-regulation of myogenin. We also present evidence that proteins from ELV-MT can be incorporated into myoblasts by using the GFP protein as cargo within ELV-MT. Taken together, our data provide a useful database of proteins from C2C12-released ELVs throughout myogenesis and reveals the importance of exosome-like vesicles in skeletal muscle biology.

## Introduction

Skeletal muscle (SkM), the largest organ in the human body, is responsible for whole-body metabolism, energy homeostasis, locomotion and serves as body protein pool. It is a highly adaptable tissue, responding to numerous environmental and physiological challenges by changing its phenotypic profile in terms of size as well as composition. During the last decade, skeletal muscle-secreted proteins have been identified and shown to play important roles in intercellular communications [1], [2], [3]. A large number of soluble peptide hormones and cytokines called myokines are capable of triggering homeostasis adaptations in other peripheral organs (*e.g.*; pancreas, adipose tissue) [4] or are involved in the process of myogenesis (*e.g*.; IL-4, IL-7 and IL-13) [5], [6]. In addition, Nishizawa et al. [7] identified a novel skeletal muscle-derived secretory factor, Musclin, whose expression was tightly regulated by nutritional changes and by insulin. Bolton et al. [3] also described that SkM of obese type 2 diabetic *Psammomys obesus* differentially secreted Periostin, an extracellular matrix protein. Periostin was further correlated with the development of cardiovascular disease associated with human obesity [8]. Furthermore, analysis of the rat skeletal muscle secretome in response to insulin [9] or tumor necrosis factor-alpha-induced insulin resistance [4] led to the discovery of numerous secreted proteins. All these data have opened an entire new field of research, placing skeletal muscle as a secretory organ.

In addition to soluble proteins and mediators, it has recently been established that cells release membrane nanovesicles called exosomes which could also mediate intercellular cross-talks under normal and pathological conditions [10]. Exosomes represent a discrete population of 30–100 nanometer-sized vesicles formed in multivesicular bodies (MVBs) during endosome maturation, by inward budding of their limiting membrane [11]. They are released from cells into the microenvironment following the fusion of MVBs with the plasma membrane. The membrane lipid composition of exosomes is similar to membrane lipid rafts, *i.e.*; they are rich in cholesterol, sphingomyelin and ganglioside GM3, conferring resistance to triton detergent and sensitivity to saponin [12]. Exosome secretion was first reported for reticulocytes during their differentiation [13]. It was then found that other hematopoietic cells (B lymphocytes, dendritic cells, T lymphocytes and mast cells) as well as non-hematopoietic cells (intestinal epithelial cells, neuroglial cells, adipocytes, myoblasts and insulinoma NIT-1 cells) also have the ability to release such nanovesicles [11]. To date, their biological functions remain largely unknown. It was suggested that they would be involved in the eradication of obsolete proteins (*e.g.*; reticulocyte exosomes contain transferrin receptor [13]) or could also play a role as modulators of the immune response [14], in the dissemination of viruses and prions, and in mediating communication between tumor cells and their microenvironment [15], [16]. Recent data indicate that exosomes might also convey information and signals between neighboring cells or distant tissues [17], [18], [19], [20] by RNA, protein and lipid transfer [21]. Indeed, the source of exosomes defines their function. For example, antigen-presenting cell-derived exosomes induce, whereas tumor-derived exosomes suppress, immune responses [22]. Thus, the characterization at the proteomic and genomic levels of exosomes released from SkM, and the study of their biological functions, would allow the identification of new potential mediators between SkM and other tissues, which could also act as endocrine signals during myogenesis.

Recently, exosome-like vesicles (ELVs) were isolated from conditioned media (CM) of C2C12 myoblasts in proliferation [23] and from human myoblast CM, 72 h after incubation in serum-free medium to induce differentiation [24]. As it is known that the skeletal muscle secretome is dynamically regulated during myoblast differentiation [1], we have postulated that muscle cells would also release different populations of ELVs during the myogenic process, and that their compositions could likely change during myotube formation. To validate this, shotgun proteomic approach was used to determine the protein contents of the nanovesicles secreted from C2C12 myoblasts and myotubes during the process of differentiation. More than 400 different proteins were identified. Using extensive bibliographic and bioinformatic analysis we found that classical components of exosomes isolated from other cell types, such as components of the ESCRT machinery of MVBs, as well as numerous tetraspanins [10] were contained in these vesicles. This suggest that C2C12 myoblasts and myotubes both secrete ELVs. Label-free quantitative proteomics revealed that their protein compositions differed in relation with the muscle cell differentiation process, revealing a tissue specific signature. We present evidence that myotube-secreted ELVs reduce myoblast proliferation and induce differentiation through, respectively the down-regulation of Cyclin D1 and the up-regulation of myogenin. Moreover, we show that proteins from ELV-MT can be incorporated into myoblasts by using the GFP protein as cargo within ELV-MT. Taken together our data reveal the importance of exosome-like vesicles for muscle myogenesis.

## Experimental Procedures

### C2C12 Culture Conditions

C2C12 mouse myoblasts (from ATCC® CRL-1772™) were routinely maintained in DMEM 4.5 g/l glucose supplemented with 10% heat-inactivated Fetal Bovine Serum (FBS), 1000 UI/ml penicillin, 1000 UI/ml streptomycin and 2 mM L-Glutamine at 37°C in humidified air containing 5% CO2. Differentiation was induced by the addition of differentiation medium (DMEM 4.5 g/l glucose supplemented with 2% Horse Serum (HS)). To isolate exosome-like vesicles from myoblast- and myotube-conditioned media for proteomic analysis and functional analysis, FBS and HS were previously centrifuged at 100,000 g overnight at 4°C. The supernatant was passed through a 0.22 µm filter and diluted with sterile DMEM.

### Isolation of Myoblast- and Myotube-secreted Nanovesicles

Myoblasts were seeded in 75 cm^2^ flasks (2500 cells/cm^2^) and grown in DMEM. When at 60% confluence, the medium was changed and myoblasts were incubated in DMEM exosome-depleted medium (DED) for 48 h. After 48 h, the conditioned medium was collected and used for ELV-MB purification. Cells were incubated in DMEM until confluence. At 100% confluence, myoblasts were grown in differentiation medium for one week. Then myotubes were incubated in differentiation medium exosome-depleted for 48 h. The conditioned medium was collected and used for ELV-MT purification.

ELVs were purified from C2C12 myoblast- and myotube-conditioned media as previously described [25]. Briefly, cell debris and organelles were eliminated at 2,000 g for 20 min and at 10,000 g for 30 min. The resulting supernatant was filtered through a 0.22 µm filter, in order to remove large particles or cellular debris (Figure S1). ELVs were pelleted by ultracentrifugation at 100,000 g for 70 min +4°C (Beckman-Coulter, Optima^tm^ L-80-XP ultracentrifuge, type 50-2Ti rotor). The nanovesicle pellet was washed with 25 ml of cold PBS. ELV protein content was quantified using Bradford protein assay. In this study, loaded exosomes are expressed as µg of protein-containing exosomes.

### Size Distribution of the Nanovesicles Secreted by C2C12 Cells

ELV size distribution was measured by photon correlation spectroscopy using a Zetasizer NanoS (Malvern Instruments, UK). An aliquot of extracted nanovesicles in PBS was analyzed at 20°C. Refractive index and viscosity of dispersant were respectively of 1.332 and 1.029 cP at 20°C [26]. Particle size distribution and corresponding mean hydrodynamic diameter were calculated by the software.

### Transmission Electron Microscopy (EM)

Nanovesicles in PBS were adsorbed on 200 Mesh nickel grids coated with formar-C. Immunogold labeling was performed by flotation of grids on drops of reactive media. Non-specific sites were coated with 1% BSA in 50 mM Tris–HCL, pH 7.4 for 10 min at RT. Antibody incubation was carried out for 4 hours at 4°C in a wet chamber with mouse monoclonal antibody raised against CD81 (sc-166028, Santa Cruz Biotechnology) (dilution 1/50) in 1%BSA, 50 mM Tris–HCL, pH 7.4. Grids were successively washed once in 50 mM Tris–HCL, pH 7.4 and pH 8.2 at RT. They were then preincubated with 1% BSA in 50 mM Tris–HCL, pH 8.2 for 10 min at RT and labeled with a goat anti mouse IgG gold-conjugated 10 nm, (Tebu bio, France) diluted 1/80 in 1% BSA-, 50 mM Tris–HCL, pH 8.2 in a wet chamber for 45 min. Grids were successively washed once in 50 mM Tris–HCL, pH 8.2 then pH 7.4 and in filtrated distilled water at RT. Grids were then floated on top of drops of silver enhancement mixture (Aurion R-GENT SE –EM) for 30 min. After washing 1 time in filtrated distilled, suspensions were colored with 2% phosphotunstic acid for 2 min and examined using a JEM Jeol 1400 transmission electron microscope (Tokyo, Japan) equipped with a Orius 600 camera (USA). Particle sizes were determined with the Digital Micrograph software.

### Western Blotting

Cells were lysed in RIPA lyses buffer (PBS, 0.1% SDS (Sodium Sodecyl Sulfate, Promega), 0.5% Sodium Deoxycholate (Sigma-Aldrich),1% Nonidet NP40 (Sigma-Aldrich), 5 mM EDTA (Ethylene Diamine Tetra Acetic Acid (Sigma-Aldrich), 1 mM Na3VO4 (Sodium orthovanadate, Sigma-Aldrich), 20 mM NaF (Sodium Fluoride, Sigma-Aldrich), 1 mM DTT (DL-Dithiothreitol, Sigma-Aldrich), cocktails of Protease inhibitors (Sigma-Aldrich)). By contrast, no treatment was applied on vesicles. Cellular and vesicle proteins were denatured in Laemmli Buffer (Tris-HCl 50 mM, Glycerol 12%, SDS 1%, beta-mercaptoethanol 4%, Bromophenol blue 0.01%, PH 6.8, (Sigma)) for 10 min at 100°C and were migrated on 10% SDS-PAGE gels (30 µg). Following electrophoresis, proteins were transferred onto nitrocellulose PVDF membranes blocked at room temperature with 4% BSA in Tris-buffered saline/0.3% Tween20 and incubated overnight at 4°C with gentle shaking with anti-CD81 (sc-166028), -Alix (sc-49268), -TSG101 (sc-6037), -TGFBRII (sc-220), -Transgelin-2 (sc-51441) antibodies from Santa Cruz Biotechnology, and anti-beta-actin (A5060) and -TSPAN 8 (SAB2102595) from SIGMA-ALDRICH, anti-Calnexin (S0998, Epitomics), total OxPhos (MS604, MitoSciences), and anti-ITGB5 (PAB11084, Tebu-Bio). All antibodies were diluted 1/1000 in 1% BSA. The signal was detected by using a horseradish peroxidase-conjugated secondary antibody (Bio-Rad, Hercules, USA) and revealed with the enhanced chemiluminescence system from Pierce (Rockford, IL).

### Protein Expression Analysis by Immunocytofluorescence

Cells were fixed in 10% formaldehyde and permeabilized with 0.1% Triton X-100. Non-specific binding sites were blocked with 1% BSA in 1x PBS for 1 h at room temperature. Cells were then incubated overnight at 4°C with specific primary antibodies (anti-Myogenin, F5D (1/50 in 1% PBS-BSA); Developmental Studies Hybridoma Bank, University of Iowa, Iowa City, IA). Detection was achieved by using Alexa 555-conjugated goat anti-mouse (1/500 in 1% PBS-BSA) (Molecular Probes/Invitrogen). Cells were mounted with Vectashield with DAPI Fluoprep mounting medium (H1200; Vector Laboratories, Peterborough, England) and examined by fluorescence microscopy using an Axiovert 200 microscope, an Axiocam MRm camera, and Axiovision 4.1 image acquisition software. The number of Myogenin positive nuclei was calculated by using the software AutoMesure from Zeiss Axiovision.

### qRT-PCR

Real-time qPCR was performed using ABsolute QPCR SYBR Green ROX Mix (Abgene, Courtaboeuf, France) with a Rotor-Gene 6000 system (Corbett Life Science, Paris, France). Data are expressed as mean±SEM. Results were normalized with the gene encoding TBP used as the reference [27]. PCR primer sequences were CCND1 (cyclin D1) S-CTTCCTCTCCAAAATGCCAG, CCND1 AS-TGGAGGGTGGGTTGGAAATG, MYOG S-CAACCCAGGAGATCATTTGC, MYOG (myogenin) AS-CATATCCTCCACCGTGATGC, TBP (TATA box binding protein) S-TTCACATCACAGCTCCCCAC, TBP AS-TGGTGTGCACAGGAGCCAAG.

### Proteomic Analyses of Exosome-like Vesicles Secreted from C2C12 Myoblasts and Myotubes

#### SDS-PAGE

exosome proteins resuspended in Laemmli buffer were stacked (2 mm) on SDS-PAGE gels (4–12% NuPAGE gels, Invitrogen) before being stained by Coomassie blue R-250 (Bio-Rad).

#### Protein digestion

Protein bands were manually excised from the gels and washed several times by incubation in 25 mM NH_4_HCO_3_ for 15 min and then in 25 mM NH_4_HCO_3_ containing 50% (v/v) acetonitrile for 15 min. Gel pieces were then dehydrated with 100% acetonitrile, incubated with 7% H_2_O_2_/7% formic acid for 15 min before being washed with the destaining solutions described above. Modified trypsin (Promega, sequencing grade) diluted in 25 mM NH_4_HCO_3_ was added to the dehydrated gel spots for an overnight incubation at 37°C. Peptides were then extracted from gel pieces in three sequential extraction steps of 15 min in 30 µL of 50% acetonitrile, 30 µL of 5% formic acid and finally 30 µL of 100% acetonitrile. The pooled supernatants were then dried under vacuum.

#### Nano-LC-MS/MS analyses

The dried extracted peptides were resuspended in 5% acetonitrile and 0.1% trifluoroacetic acid and analyzed by online nanoLC-MS/MS (Ultimate 3000, Dionex and LTQ-Orbitrap XL, Thermo Fischer Scientific). Peptides were sampled on a 300 µm × 5 mm PepMap C18 precolumn and separated on a 75 µm × 150 mm C18 column (Gemini C18, Phenomenex). The nanoLC method consisted in a 120-minutes gradient ranging from 5% to 40% acetronitrile in 0.1% formic acid at a flow rate of 300 nL/min. MS and MS/MS data were acquired using Xcalibur (Thermo Fischer Scientific). Spray voltage and heated capillary were respectively set at 1.4 kV and 200°C. Survey full-scan MS spectra (m/z = 450–1600) were acquired in the Orbitrap with a resolution of 60,000 after accumulation of 10^6^ ions (maximum filling time: 500 ms). The five most intense ions from the preview survey scan delivered by the Orbitrap were fragmented by collision induced dissociation (collision energy 35%) in the LTQ after accumulation of 10^4^ ions (maximum filling time: 100 ms).

#### Peptide and protein identifications

RAW files were processed using MaxQuant [28] version 1.3.0.3. Spectra were searched against the Uniprot database (August 2012 version, Mus musculus taxonomy 10090, 86644 sequences, Bos taurus taxonomy 9913, 34280 sequences and Equus caballus taxonomy 9796, 24299 sequences) and the frequently observed contaminants database (notably containing protein sequences from serum proteins) embedded in MaxQuant. Trypsin was chosen as the enzyme and 2 missed cleavages were allowed. Precursor mass error tolerances were set respectively at 20 ppm and 6 ppm for first and main searches. Fragment mass error tolerance was set to 0.5 Da. Peptide modifications allowed during the search were: trioxidation (C, fixed), acetyl (N-ter, variable), dioxidation (M, variable), oxidation (M, variable) and deamidation (NQ, variable). Minimum peptide length was set to 7 amino acids. Minimum number of peptides, razor+unique peptides and unique peptides were set respectively to 2, 2 and1. Maximum false discovery rates - calculated by employing a reverse database strategy - were set to 0.01 at peptide and protein levels. Raw MS data files, unfiltered protein groups and peptides tables are available at ProteomeXchange (www.proteomexchange.org, accession PXD000022).

#### Data analysis

Proteins identified as “contaminants” (*i.e*.; present in the frequently observed contaminants database embedded in MaxQuant) or “reverse” (*i.e.*; present in the reverse database used for false discovery rate calculations) were discarded from the list of identified proteins. Proteins identified in the bovine and/or horse databases but not in the mouse one were also deleted. Proteins noted as lying in plasma in Uniprot knowledgebase were also discarded from this list. Finally, only proteins identified in 2 biological replicates with a minimum of 2 spectral counts (SC) in 1 biological replicate were considered as member of exosomes from myoblasts and/or myotubes.

For quantitative comparison of ELV-MB and ELV-MT proteomes, we used a beta-binomial test specifically developed to test the significance of differential protein abundances expressed in SC [29]. To be considered as significantly enriched in one type of ELV compared to the other, a protein must have been found positive to the test (at 95% confidence level), exhibit a total SC ≥5 in one type of ELV and, if identified in both types of ELV, present an enrichment ratio ≥5.

### Impedance Measurement with the xCELLigence RTCA DP Instrument (Roche)

To monitor the effect of C2C12 ELVs on the C2C12 proliferative capacities, we used the xCELLigence live cell analysis System (Roche Applied Science) which offers dynamic live cell monitoring [30]. The System measures electrical impedance across interdigitated micro-electrodes integrated on the bottom of tissue culture E-Plates. Background of the E-plates was determined in 50 -µl medium and subsequently 150 -µl of the C2C12 cell suspension was added at optimal seeding number (2500 cells/cm^2^). E-plates were placed into the Real-Time Cell Analyzer (RTCA) station. One day after plating, cells were grown in DMEM 4.5 g/l glucose supplemented with 5% exosome-depleted FBS, and incubated with 2 µg ELVs collected either from myoblasts or myotubes, and monitored again every 15 min for 22 h. The impedance measurement provides quantitative information about the biological status of the cells, including cell number, viability, and morphology. Impedance was represented by the cell index (CI) values ((Z_i_-Z_0_) [Ohm]/15 [Ohm]; Z_0_: background resistance, Z_i_: individual time point resistance) and the normalized cell index was calculated as the cell index CI_ti_ at a given time point divided by the cell index CI_nml-time_ at the normalization time point (nml_time). At the end of the experiment, cells were trypsinized, counted and their size was determined by using the Scepter 2.0 handheld automated cell counter from Millipore. We used the 60 µm sensor to obtain size distributions between 6 and 36 µm.

### Production of Exosomes Expressing GFP

Non replicative adenoviruses expressing the green fluorescent protein (GFP) were generated by homologous recombination in *Escherichia coli* BJ 5183, as previously described [31]. Co-transformation of *E. Coli* BJ5183 led to recombination between GFP cloned in pCNA3 and a viral vector recombinogenic with the pCDNA3 cytomegalovirus promoter and poly(A) sequence (VmcDNA, provided by S. Rusconi, University of Fribourg, Switzerland). Recombinants were screened by PCR with pair of primers that annealed to portion of the CMV promoter which is brought in by homologous recombination (5′-GACGGATGTGGCAAAAGTGA-3′ and 5′-ATGGGGTGGAGACTTGGAAATC-3′). Positive clone harboring GFP was further amplified in *E. coli* XL-1 Blue, digested with PacI, and transfected by the calcium phosphate method into HEK-293T cells (ATCC® CRL-11268™) to produce viral particles. Adenovirus were purified by ultracentrifugation on CsCl gradient and stored in PBS and 10% (v/v) glycerol at –80°C. Viral titer of stocks was 5.6×10^10^ particles/ml.

Differentiated C2C12 cells (myoblasts seeded at 2500cells/cm^2^ in 75 cm^2^ flasks) were infected with GFP expressing adenovirus for 24 h in DMEM 4.5 g/l glucose supplemented with 2% HS at 37°C (1.6 µl of adenovirus per 75 cm^2^ flask). After 24 h, all myotubes had green fluorescence in the cytoplasm indicating that all cells had been infected by the adenovirus. Myotubes were washed with PBS in order to remove both non integrated adenovirus and exosomes from serum, and were incubated for another 48 h in exosome-depleted DMEM. ELV-MT-GFP accumulated in conditioned medium for 48 h were extracted as described above.

C2C12 myoblasts were seeded in 6-well plates at 2500 cells/cm^2^. When at 80% confluence, myoblasts were incubated with 2 µg ELV-MT-GFP per ml of medium. Twenty-four hours later, the medium was removed and cells were visualized with Zeiss Axiovert 200M Fluorescence/Live cell Imaging microscope equipped with the Axiovision software.

### Statistical Analyses

Statistics analyses were performed using SPSS 13.0 software. All results were expressed in mean +/− standard error of the mean (SEM). Parametric Student *t*-test was used for mean comparison, a *p* value <0.05 was considered significant. ANOVA one way test was applied to determine the effect of ELVs treatment on cell death, cell size, and cell proliferation. Chi-square test was used to determine whether the % of myogenin-positive nuclei was significantly higher 48 h post-differentiation, when myoblasts were treated with ELV-MT compared to ELV-MB. *p*-values <3.84 (considering 1 degree of freedom) indicated that the percentages are significantly different.

## Results

As exosomes are present in all serums used for cell culture, it was necessary to remove the exosomal fraction from fetal bovine and horse serums to avoid contaminations of C2C12 myoblast- and myotube-conditioned media. Thus, we verified that these new serum compositions did not affect C2C12 cell growth. As shown in Figure S2, growth for 48H in 10% fetal bovine exosome-depleted serum neither affected proliferation nor C2C12 myoblast cell sizes. C2C12 myotube formation was also not affected when cells were incubated in 2% horse exosome-depleted serum compared with normal serum (Figure S3).

### C2C12 Myoblasts and Myotubes Release Nanovesicles with Exosome-like Properties

**Figure 1:**
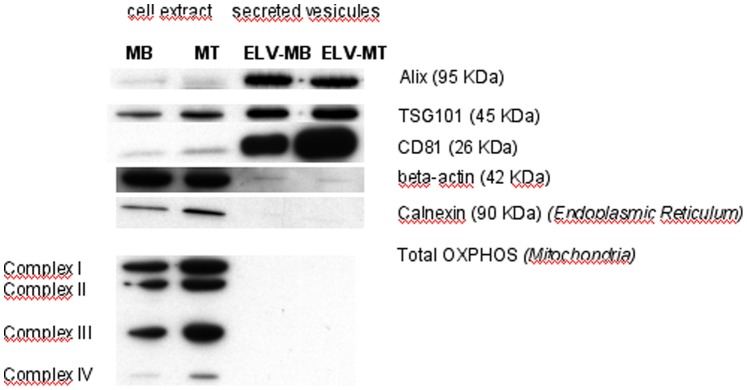
Quality analysis of purified ELV preparations by Western-blot. Equal protein amounts of extracts prepared from cells or exosomes were subjected to western blot analysis. The multivesicular body markers TSG101 and Alix (*ALG2-interacting protein 1*), and the tetraspanin CD81, were strongly enriched in exosome preparations compared with cell lysates.

**Figure 2:**
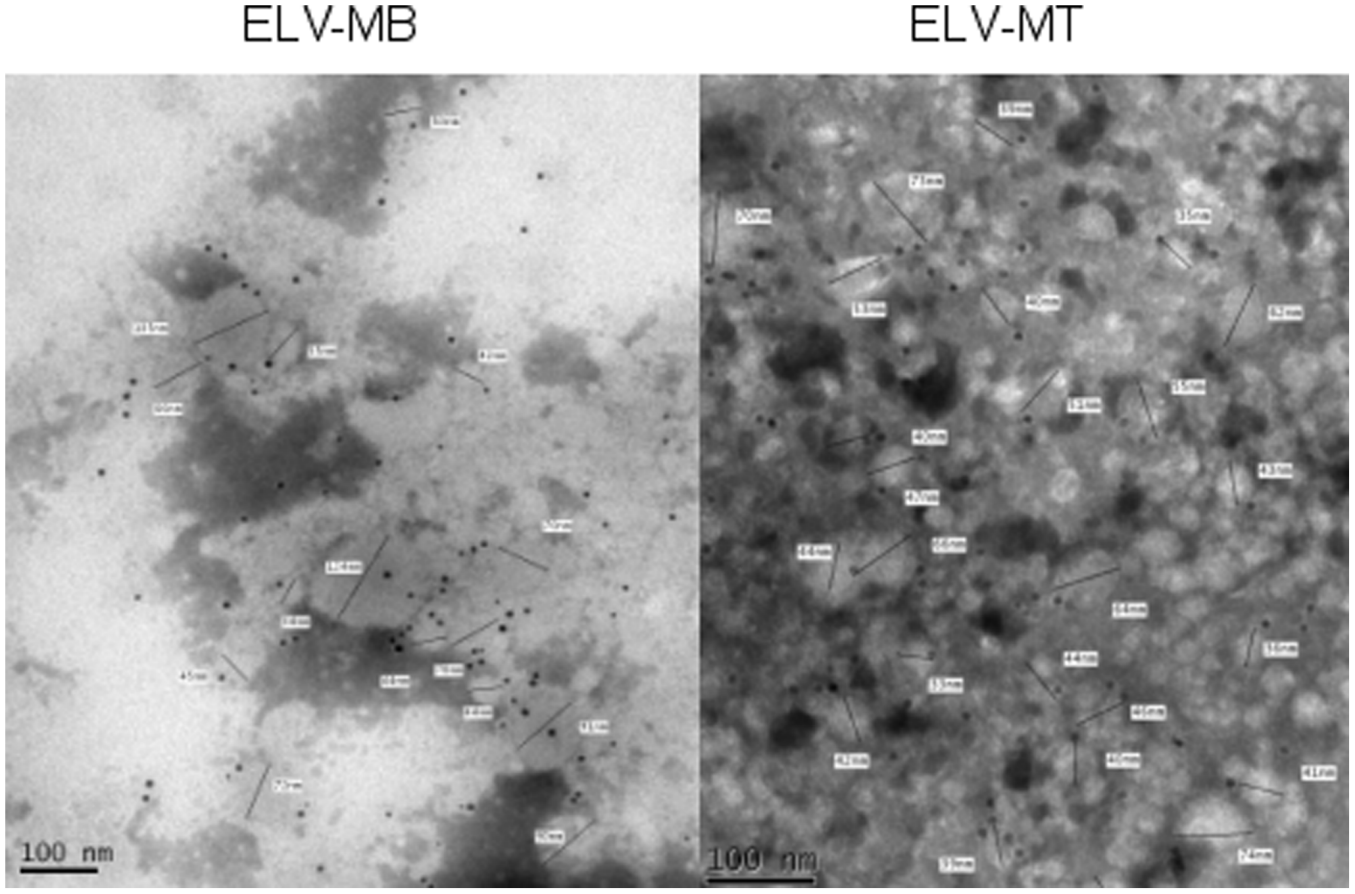
Transmission electron microscopy images of purified nanovesicles from myoblasts (ELV-MB) or myotubes (ELV-MT) conditioned media. Bar = 100 nm. Nanovesicles are labeled with anti-CD81 gold particles specifically expressed at exosome-like vesicle membranes.

Electron microscopy and dynamic light scattering analyses were performed on the nanovesicle pellets obtained after ultracentrifigation of C2C12 conditioned media. Three main types of vesicles are released by cells: apoptotic bodies (500 nm–3 µm in diameter) released by cells undergoing apoptosis; shedding microvesicles that bud from the plasma membrane (100 nm–1 µm) and exosomes that are released by exocytosis from multivesicular bodies of the endosome (stated variously as 30–100 or 30–150 nm) [32], [33]. The size distribution of such particles in our preparations was measured by photon correlation spectroscopy. Representative distributions for myoblast and myotube cell-derived vesicles are shown in Figure S1. In each case 3 separate preparations were analyzed with very similar results. No particles of >500 nm were detected in any preparation (Figure S1) thus excluding apoptotic bodies as components of the samples. The vesicle size distribution displayed a bell-shaped curve, suggesting a homogeneous population in agreement with the reported size of exosomes (Figure S1) [26], [32], [33] and Western-blot analysis on the nanovesicle pellet showed a strong enrichment of classical exosomal proteins compared with the parent cells (Figure 1). These nanovesicles expressed exosomal proteins at their membrane surface such as CD81 (Figure 2). As previously noticed for other conventional markers of exosomes in other cell types (*e.g.*; CD63, CD9), we found that CD81 is observed on vesicles of various sizes, indicating that multivesicular endosomes in muscle cells contain intraluminal vesicles of heterogeneous sizes (Figure 2).

Proteomic analyses were performed on 3 independent preparations of ELVs, each resulting from the ultracentrifugation of 200 ml of conditioned media, filtered through a 0.22 µm filter, from either 60–70% confluent C2C12 myoblasts or fully differentiated C2C12 myotubes. Both ELV preparations were solubilized in SDS-PAGE sample buffer and stacked on SDS-PAGE gels. Gel bands were excised, contained proteins were submitted to trypsin digestion and extracted peptides subjected to nanoLC-MS/MS analysis. Only proteins identified in 2 biological replicates with at least SC≥2 in one of them, were considered as present in ELVs. Protein database searching of MS/MS data resulted in the identification of 455 unique proteins in ELVs (Table S1). Among these, 334 and 383 were found in ELVs released from myoblasts (ELV-MB) and myotubes (ELV-MT), respectively. Consistent with the proposed late endosome origin of exosomes, several identified proteins were associated with multivesicular body biogenesis (*i.e.*; CHMP4B, BROX, FAM125A, FAM125B, LAMP1, LAMP2, TSG101, VAMP2, VAMP3, VAMP5, VPS28, VPS35, VPS37B, VPS37C) (Table S1). Furthermore, muscle ELVs contained 20 of the 25 proteins that are often identified in exosomes from various origins (*i.e.*; HSPA8, CD9, GAPDH, CD63, CD81, ANXA2, ENO1, EEF1A1, PKM2, YWHAE, PDCD6IP, YWHAZ, EEF2, LDHA, HSP90AB1, ALDOA, MSN, ANXA5, PGK1, CFL1) [34].

Among the 163 proteins previously identified in ELVs released from C2C12 myoblasts using a similar proteomic analysis [23], 71 were also included in our list of 334 proteins from ELV-MB (Table S2), leaving 263 that had not formally been associated with C2C12 myoblast ELVs. Recently, Le Bihan et al. identified 564 proteins within human myotube-derived ELVs [24]. Among these, 238 were also included in the list of 455 unique proteins in murine ELVs (Table S2).

**Figure 3:**
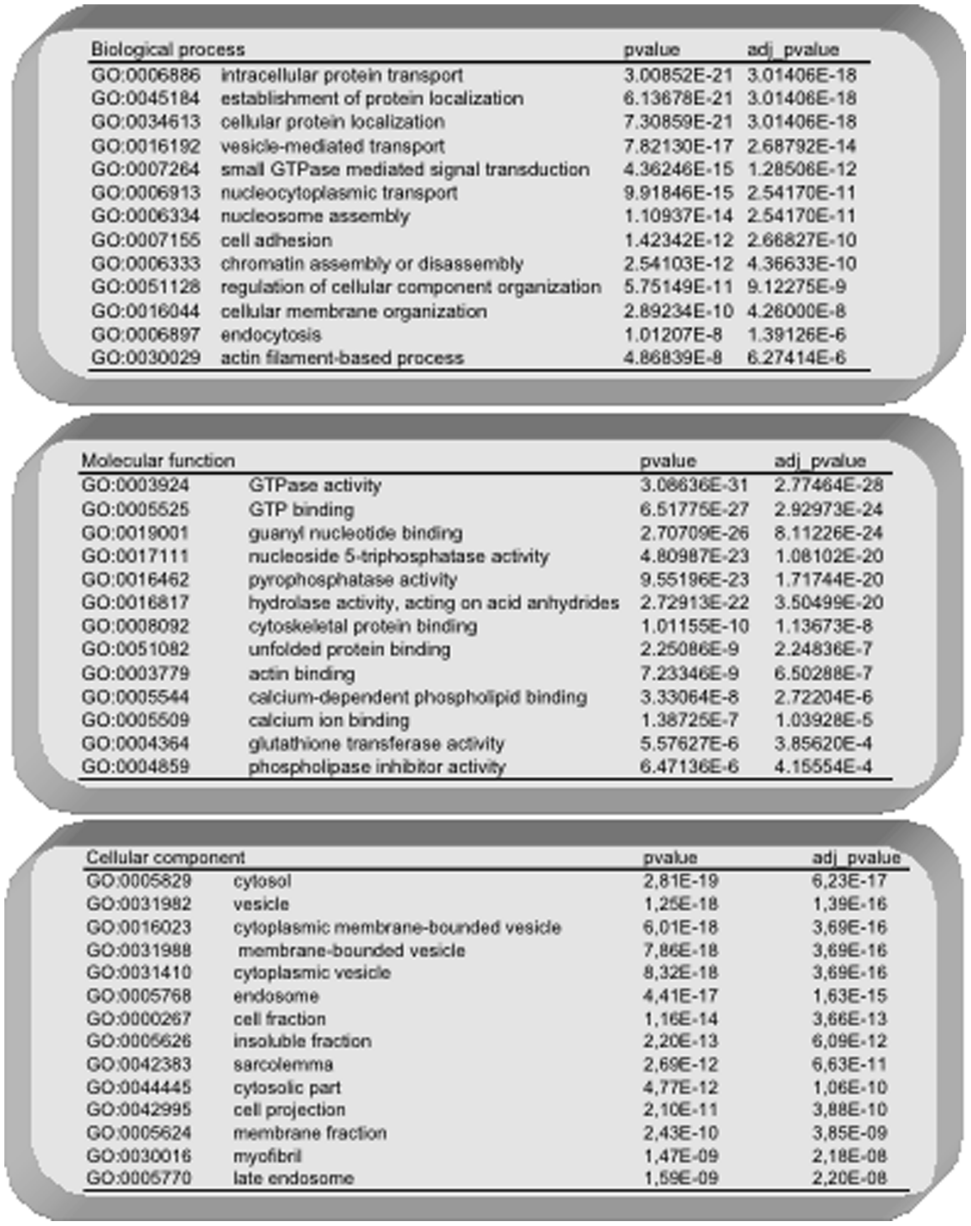
Functional analysis of ELV-MB and ELV-MT common proteins using Babelomics 4.0. Only significant Gene Ontology terms are indicated.

In order to have a functional overview of ELV-MB and ELV-MT proteins, we used the integrative platform Babelomics (http://babelomics.bioinfo.cipf.es) [35] to determine significant over-representation of Gene Ontology (G.O.) functional annotations, by single enrichment analysis. The 455 unique proteins in ELVs were analyzed. When considering an adjusted *p*-value<0.01, 35 significant G.O terms were found (Figure 3). Significant G.O. terms for ‘biological processes’ contained proteins involved in endocytosis and intracellular transport and localization, cell adhesion, small GTPase mediated signal transduction, DNA packaging, and cytoskeleton organization. Significant G.O. terms for ‘molecular functions’ were GTPase activity, calcium ion binding, pyrophosphatase and hydrolase activity, unfolded protein binding and cytoskeleton binding. The proteins included in the top 10 significant G.O. terms for ‘cellular components’ were located in vesicles, early and late endosomes and sarcolemma. Thus, taken all together, these results support the conclusion that the nanovesicles secreted in the extracellular medium of C2C12 cells consist largely of exosomes.

To determine whether C2C12 ELVs expressed particular protein subsets compared to the whole C2C12 secretome, we compared the lists of 455 unique proteins in ELVs from C2C12, with the one of 635 proteins previously identified in C2C12 myoblast secretome [1]. Among the 635 secreted proteins, 84 and 98 were also identified in ELV-MB and ELV-MT, respectively. The weak overlapping between the datasets confirmed that C2C12 ELV proteins identified in this study were mainly contained, or at least strongly enriched, in the nanovesicles collected from C2C12 conditioned media.

Functional enrichment analysis by comparing the set of 635 secreted proteins from myoblasts with the set of 334 proteins from ELV-MB, revealed a significant enrichment in ‘nucleoside-triphosphatase activity’ (GO :0017111, *p* = 4.108^−30^), ‘cell part’ (GO :0044464, *p* = 8.667^−36^) and ‘transport’ (GO :0006810, *p* = 7.607^−25^) in the list of ELV-MB proteins. In addition, none of the GO categories previously found as significantly enriched in proteins from ELV-MT and ELV-MB (Figure 3) were found in the set of 635 secreted proteins [1]. By contrast, the 635 secreted proteins were significantly enriched in genes coding for ‘cytokines’ when compared with the set of 334 ELV-MB (KEGG pathway mmu05322, *p* = 0.0006767). These data indicate that skeletal muscle probably uses distinct pathways of secretion for distinct protein subsets.

### Myoblasts and Myotubes C2C12 Release Specific ELVs with Distinct Protein Compositions

**Figure 4:**
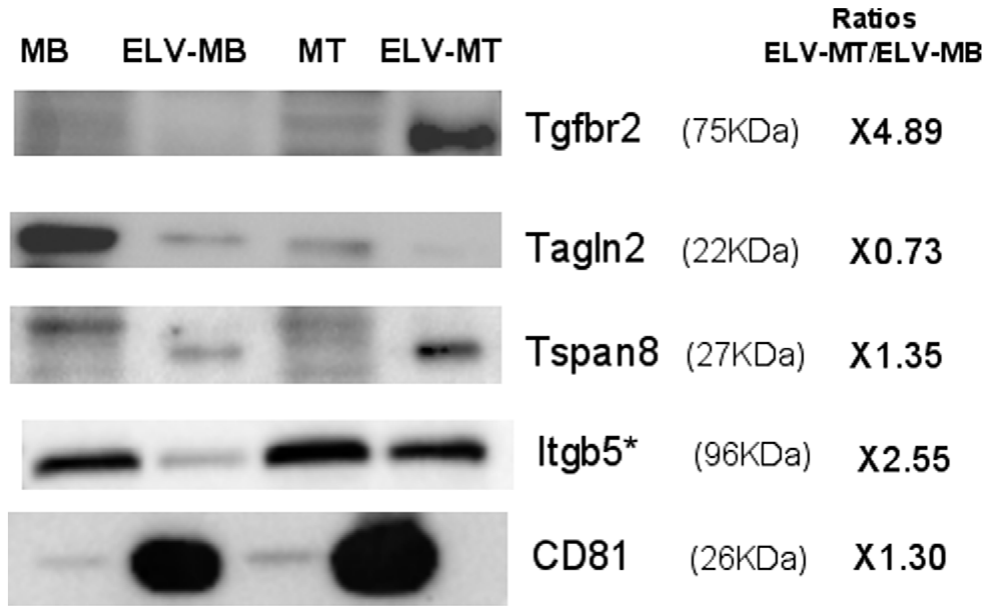
Western-blot analysis of proteins found differentially expressed between ELV-MB and ELV-MT (see Table S1). ITGB5 (Itgb5) : Integrin beta-5; TGFBR2 (*Tgfbr2*) : Transforming growth factor, beta receptor II; TAGLN2 (Tagln2) : Transgelin-2; TSPAN8 (*Tspan8*) : Tetraspanin-8. *, only detected in ELV-MT but not selected as statistically more expressed in ELV-MB (see Table S1).

Previous proteomic analyses and microarray-based studies have identified differential waves of protein and mRNA expressions across the early, mid, and late stages of C2C12 differentiation, suggesting their roles in myogenesis [36], [37]. In this study, using label-free quantitative proteomics, we also detected differential expression of 31 and 78 proteins respectively in ELV-MB and ELV-MT, indicating that during myogenesis, ELV protein content is also regulated (Table S1, Figure 4, Figure S4). Among these proteins, 4 proteins previously identified only in the proteome of differentiated myotubes [36] were also detected only in ELV-MT (*i.e.*; SGCA, DAG1, MYH1, MYH4). In addition, 22 muscle-specific proteins were identified (*i.e.*; ATP2A1, ATP2A2, CAMK2G, CAPZB, CASQ1, CASQ2, CRYAB, DAG1, DES, FLNC, ITGB1, NES, RHOA, SGCA, SGCD, SNTB1, SNTB2, TLN1, TTN, UTRN, VCL and VIM) (http://wiki.geneontology.org/index.php/Muscle_Biology) showing that ELV composition partially reflects their muscle origin.

Bioinformatic analysis revealed that the 78 proteins more significantly present in ELV-MT were enriched in genes involved in muscle contraction (GO :0006936, *p* = 0.00005457) and metal ion transmembrane transporter activity (GO :0046873, *p* = 0.000007549) when compared to the proteins common to ELV-MB and ELV-MT. No significant functions were found for the group of 31 specific ELV-MB proteins.

### ELV-MT Reduce Myoblast Proliferation

The effects exerted by conditioned media (CM) on the development of muscle cells have been documented for a long time and it has been demonstrated that myogenic cells modify their own extracellular media by secreting factors that exert autocrine and paracrine effects on the differentiation process [38]. Furthermore, it is well-established that CM from myotubes exerts dramatic effects on pre-myogenic cells [39], [40]. To investigate whether ELV-MT and ELV-MB might also participate in these processes, myoblasts were grown in DMEM depleted-exosomes complemented either with ELV-MB or ELV-MT (2 µg/ml of medium). This concentration was chosen after previous published experiments had shown effects of exosomes in concentrations varying from 10 to 1000 µg/ml [17], [18]. We found that C2C12 released 0.423±0.0997 µg/ml ELVs per 24 h, in exosome-free medium. In order to detect the biological effect of ELV-MT on myoblasts it was thus necessary to use higher quantities of ELVs, but compatible with a physiological effect. We have decided to use 2 µg ELV-MT, which is less than the previous studies demonstrating the biological effect of exosomes, and less than the concentration of exosomes detected in plasma [41].

**Figure 5:**
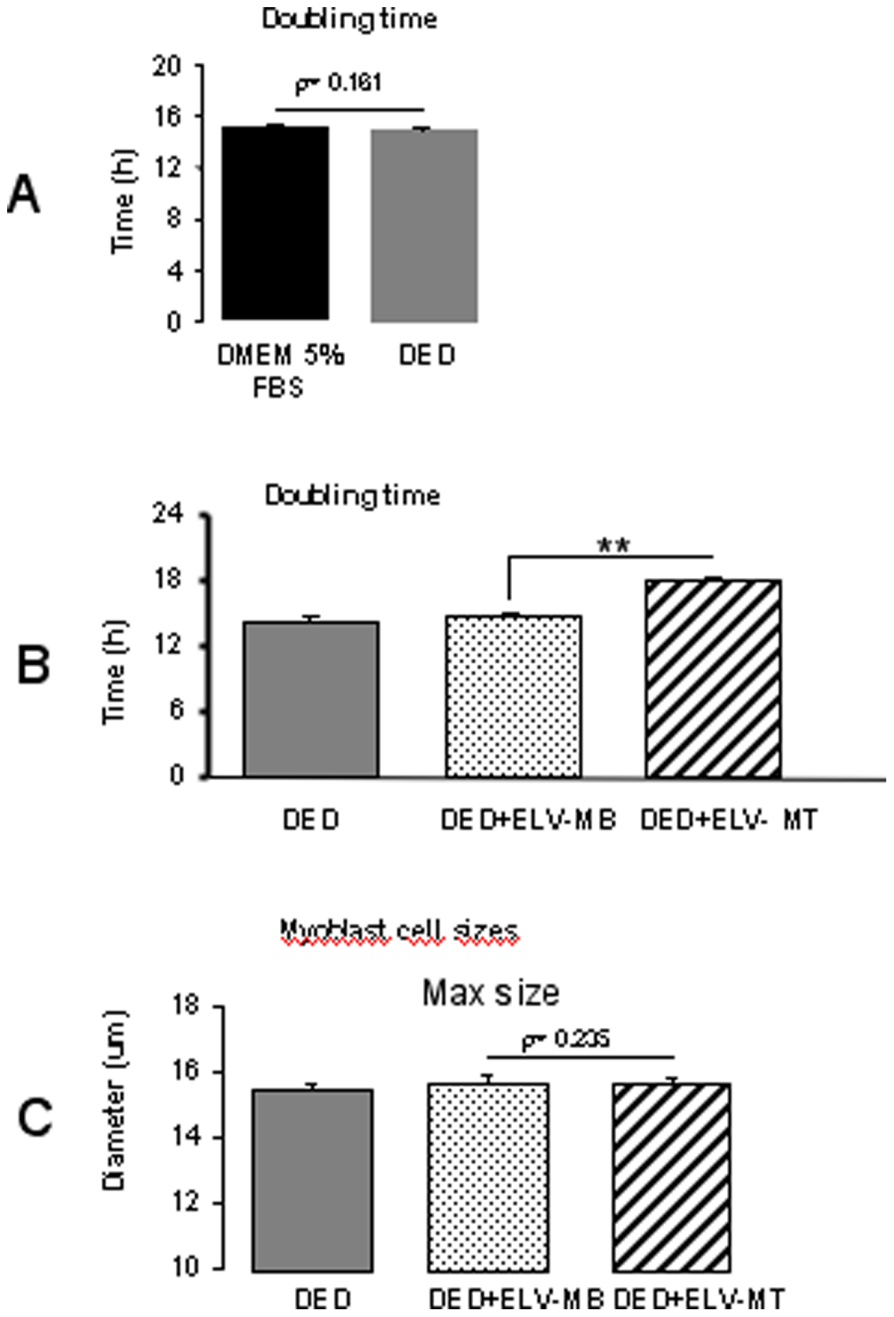
Effects of ELV-MT on myoblast proliferation. (A)_Myoblasts were grown in 96-well plates either with DMEM 5% FBS or with DMEM 5% Exosome-Depleted serum (DED) in order to calculate the cell index doubling time, using the xCELLigence RTCA HT Software. The doubling time is the time required for cell index to double and thus represents time when whole cell population has performed at least one division. As shown, C2C12 myoblasts divided once in both control media, every 15 hours (replicates = 8). B_Myoblasts were incubated with DED supplemented either with 2 µg of ELV-MB or 2 µg of ELV-MT/ml of medium. C2C12 doubling time in each medium are shown (replicates = 8). (** = *p*-values<0.05, DED+ELV-MB *vs* DED+ELV-MT). C_24 h after treatment with ELVs, C2C12 myoblasts were trypsinized and resuspended in DED for size determination by the Scepter 2.0 handheld automated cell counter. Cells were diluted in 100 µl DED in order to analyze at least 10,000 cells/ml for each replicate as recommended by the supplier (replicates = 8).

**Figure 6:**
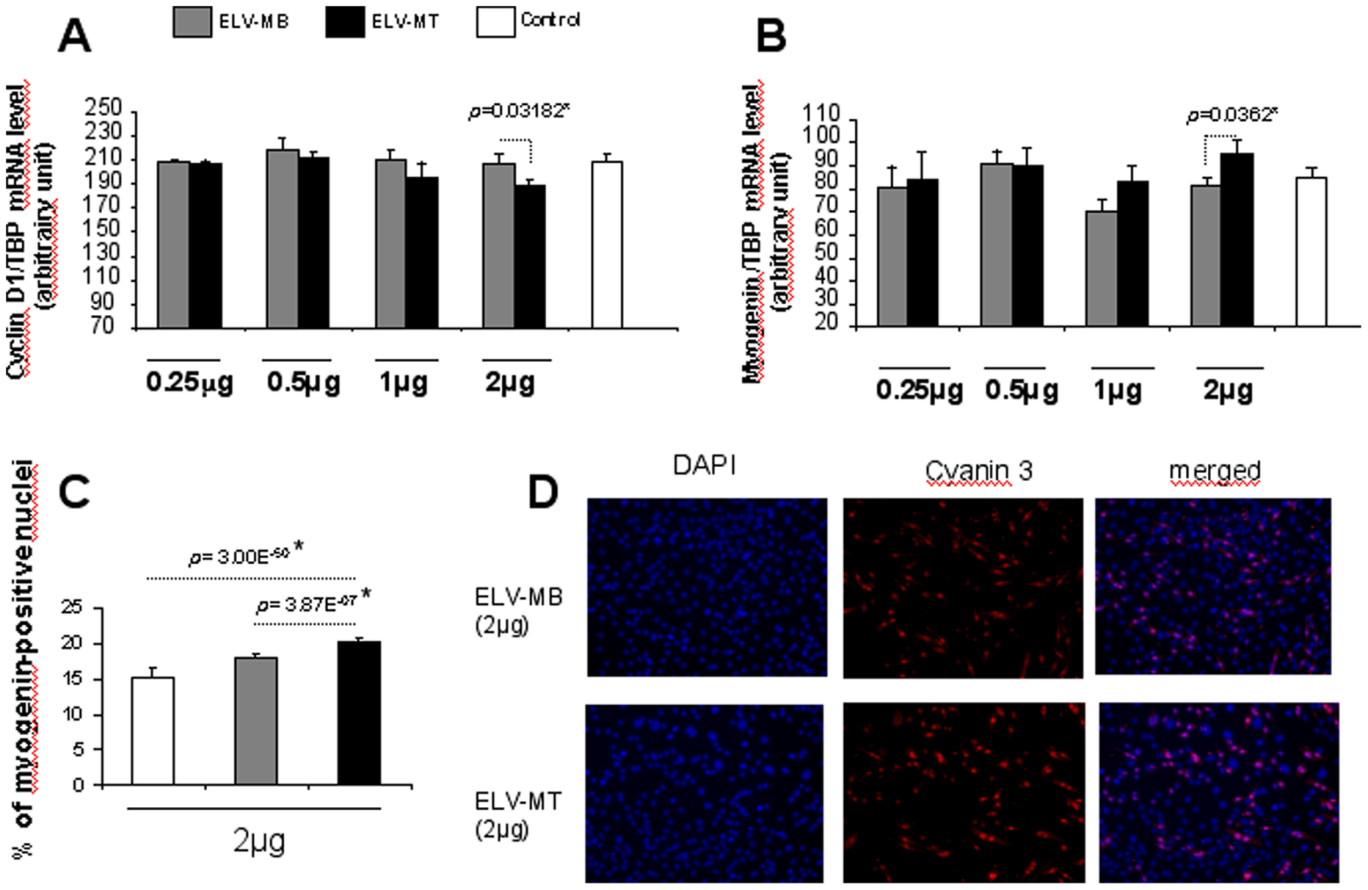
Effects of ELV-MT on myoblast cyclin and myogenin expressions. A_mRNA levels of CCND1 in C2C12 myoblasts grown in DED supplemented with ELV-MB or ELV-MT (n = 5 replicates). B_mRNA levels of Myogenin 48 h after the induction of C2C12 differentiation (n = 5 replicates). All qrt-PCR values are expressed as means ± SEM (* = *p*<0.05); C_ Myoblasts were pre-treated with ELVs during proliferation (2 µg/ml of medium). Then the percentage of C2C12 nuclei expressing myogenin was quantified by immunocyofluorescence, 48 h after the induction of C2C12 differentiation (n = 5 replicates). Chi-square test was used to determine whether the % of myogenin-positive nuclei was significantly different. (*) *p*-values <3.84 (considering 1 degree of freedom) are significant. D_ Representative pictures of the myogenin staining by using 2 µg ELVs.

The cell growth curves were automatically recorded every 15 minutes for 24 hours on the xCELLigence System in real time, and the cell doubling time was calculated. As shown in Figure 5A, the cell doubling time was 15 h in normal growth medium (DMEM or DMEM exosome-depleted) and we used this time as a reference to calculate the doubling time of the cells incubated either with ELV-MB or ELV-MT. As indicated in Figure 5B, changes in the cell index doubling time depended on the origin of C2C12 ELVs. ELV-MT had an anti-myoblast cell proliferation effect, as determined by a significant increase in cell doubling time. ELV-MB had no effect compared with the control medium. In addition, cell cycle analysis by flow cytometry, showed that myoblasts incubated with ELV-MT had a higher number of cells in the G1 phase compared to when incubated with ELV-MB (Figure S5), confirming the effect of ELV-MT on myoblast proliferation. All these effects on proliferation were neither associated with modifications of cell morphologies, as determined by light-microscopy (Figure S6) nor of mean cell sizes, as calculated with the Scepter 2.0 cell counter (Figure 6A). In addition, treatment with ELVs did not significantly increase cell death compared with control medium (Figure S6 and Figure S7).

In an attempt to unravel the mechanisms underlying the cell growth effect of ELV-MT, we quantified the expression of Cyclin D1 gene (CNND1) involved in the regulation of the cell-cycle [42]. Myoblasts incubated with ELV-MT displayed lower level of Cyclin D1 mRNA when compared with cells incubated with ELV-MB (Figure 6A).

### C2C12 ELV-MT Induce Myoblast Differentiation

In many cell lineages, arrest of proliferation induces differentiation or apoptosis. In the case of muscle cells, cell cycle exit and differentiation are coupled during myogenesis [43]. Expression of myogenin is considered one of the earliest molecular markers for cells committed to differentiation *in vitro* and is a prerequisite for efficient myofibers formation and muscle gene expression. As incubation of C2C12 myoblasts with ELV-MT during proliferation slowed down cell growth, we postulated that ELV-MT would be involved in the commitment of myoblasts in the process of differentiation. C212 myoblasts were treated once with either ELV-MB or ELV-MT until confluence. Myogenin mRNA level was quantified 48 hours after incubation in the differentiation medium. As shown in Figure 6B, myogenin expression was significantly higher in cells incubated with ELV-MT during proliferation compared with those grown with ELV-MB. We also analyzed myogenin expression at the protein level by counting the number of myoblast nuclei expressing myogenin (Figure 6C and Figure 6D). As shown in Figure 6C, the percentage of positive nuclei for myogenin was significantly higher in cells incubated with ELV-MT during proliferation, compared with those incubated with ELV-MB (2 µg/ml of medium). These results indicate that ELV-MT are involved in induction of early markers of differentiation.

### C2C12 ELV-MT Can Transfer Proteins from Myotubes to Myoblasts

**Figure 7:**
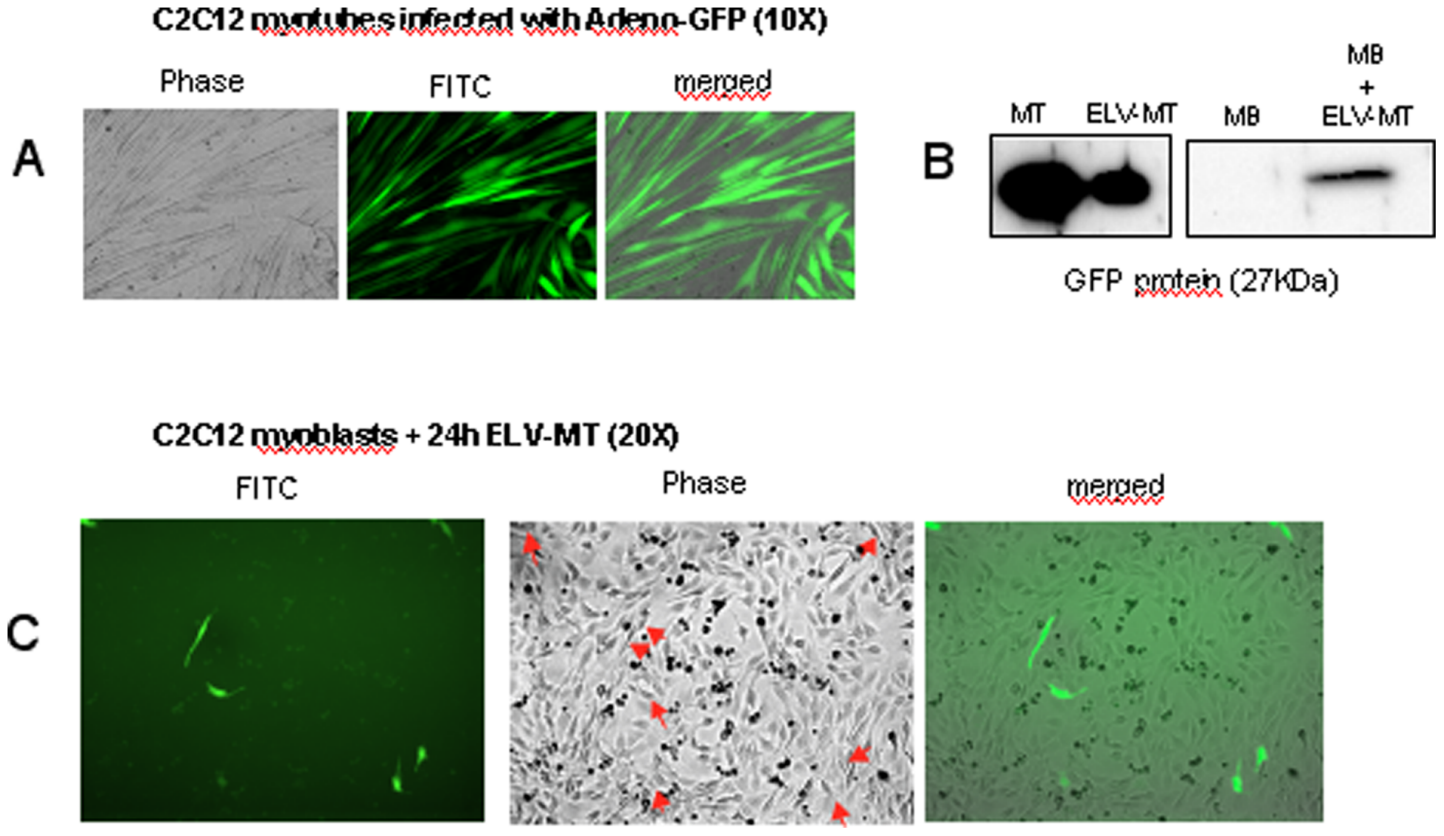
ELV-MT can transfer cytoplasmic GFP proteins from myotubes to myoblasts. A_ Differentiated myotubes infected with a non-replicative adenovirus expressing GFP protein. B_ Western-blot analysis to detect GFP protein in infected myotubes (1 µg), their released ELV-MT (1 µg), and in MB incubated with GFP-containing ELV-MT (60 µg). B_Myoblasts were incubated for 24 h with GFP-containing ELV-MT released from myotubes (2 µg/ml of medium). Arrows indicate the cells which express GFP in cytoplasm.

In order to determine whether ELV-MT could transfer their contents into myoblasts, C2C12 myotubes were infected with a non replicative adenovirus expressing the GFP protein. After 48 h post-infection, all myotubes expressed the GFP protein in the cytoplasm (Figure 7A and Figure 7B). ELV-MT released from infected MT, were collected and used to treat C2C12 myoblasts at 80% confluence. After 24 h, a fluorescent signal was detected in the cytoplasm of myoblasts, indicating that GFP had been transferred (Figure 7B and Figure 7C). This result confirms the ability of myoblasts to uptake ELV-MT from myotubes and suggests a potential role of some ELV-MT proteins in regulating myoblast proliferation.

## Discussion

Until now, myokines from muscle-cell secretome provided a conceptual basis to explain how muscles communicate to other organs [4]. In this study, we show that C2C12 cells secrete nanovesicles with exosome-like properties (ELVs) which are involved in the process of differentiation. Moreover, we provide a useful database of proteins from C2C12-released ELVs throughout myogenic proliferation and differentiation.

Discovered nearly 30 years ago, the earliest role proposed for exosomes was to shed unwanted proteins from cells undergoing terminal differentiation [44]. Although this perspective may apply in certain situations, like within the nervous system [45], the protein composition of exosomes also seems consistent with a positive role in communication with other cells [18], [32], [46], [47]. In this study, we found that C2C12 ELVs expressed specific cell-adhesion molecules on their surfaces (*e.g.*; ITGB1, NCAM, CD9, CD81, CD44, Myoferlin) which are involved in the recognition and adhesion of competent myoblasts during the process of myoblast fusion [48], [49], [50], [51], [52]. As some of them are regulated in ELVs during myogenesis (*e.g.*; TSPAN8), they could facilitate ELVs uptake by myoblasts. Indeed it was demonstrated that TSPAN8, present in our study, and mainly expressed in ELV-MT, is involved in exosome uptake [53], [54], [55].

Interestingly, the majority of the proteins identified in this study were found both in ELV-MB and ELV-MT preparations indicating that C2C12 ELVs composition remains quite constant, even though the organization of the cellular organelles and the plasma membrane of myoblasts change dramatically with the consequent formation of a single functional unit. This result further supports the concept that sorting of proteins into C2C12 ELVs seems to be highly selective. We also found that ELV-MB and ELV-MT have specific protein subsets, in relation with the myogenic process, that could sustain part of their biological effects together with other exosome-containing molecules like mRNA and miRNA. Previous studies have demonstrated that exosomes can transfer their miRNA and mRNA contents into recipient cells. For example, Montecalvo and colleagues have demonstrated that dendritic cells (DCs) release exosomes that are loaded with distinct sets of miRNAs, dependent on the status of DC activation [56]. They provide proof of principle that, after being transferred by exosomes, miRNAs can repress mRNAs in target cells [56]. Transfers of specific mRNA between exosomes and target cells have also been described [18], [20]. Recently, it was shown that human myoblast-released ELVs also contained genomic information [24]. In this study, by using GFP protein as cargo, we provide evidence that transfer of proteins can occur between myoblasts and myotubes and, in addition to miRNA and mRNA, could be an additional mechanism in the control of the recipient cells.

As previously found [23], muscle ELVs contain proteins of the G-protein family. They are involved in many cellular processes [57] including myogenesis [58]. As it was shown that G-proteins remain functionally active in exosomes [59], it would be interesting to determine whether they could play a role during the process of C2C12 differentiation. In addition, it has also been described that cells export proteins involved in specific signaling pathways in order to reduce their intracellular concentrations, and that it would represent a novel mechanism for signaling attenuation [60], [61]. For example, CD9 and CD82 boost the release of exosomes containing ß-catenin, thereby reducing cellular levels of ß-catenin and inhibiting the Wnt pathway. On the other hand, cells lacking CD9, produce fewer exosomes and show higher Wnt signalling activity [60]. It was also discovered that the release of LMP1 *via* exosomes, chaperoned by CD63, strongly reduces LMP1-mediated NFkappaB signaling [61]. Further studies are now needed to identify which part of ELV-MT composition is responsible for their actions on myoblast proliferation and differentiation.

Our results also support a model in which ELV-MT may generate endocrine signals during myogenesis. Our data shows that ELV-MT induced myoblast growth arrest and committed cells to differentiate. Effects of exosomes on cell proliferation have been previously reported for other cell types. For example, dendritic cell-derived exosomes trigger proliferation of natural killer cells [62] whereas exosomes released from cells of the thymus suppress the proliferation of CD4^+^CD25^−^T cells [63]. Exosomes derived from cancer cell lines increase proliferation of the releasing cells [64]. Our data extend these observations by suggesting that this effect would be part of the normal process of myogenesis, likely to coordinate myoblasts during the differentiation step. Although a direct physiological role for muscle exosome-like vesicles has yet to be demonstrated, our data indicate that they could participate in the dialog between myoblasts and myotubes, probably in combination with myokines [5].

From a patho-physiological point of view, used as a molecular signal that accelerates myogenesis, muscle ELV treatment might be useful to ameliorate muscle diseases or to facilitate recovery from muscle atrophy and/or injury. Additional studies are now required to further determine the exact role of ELVs from skeletal muscle cells in tissue morphogenesis and in intercellular communication occurring in complex pathologies like muscle insulin-resistance associated with type 2 diabetes. Moreover, complementary experiments should address the question of the underlying mechanisms of protein sorting into ELVs. The inter-relationships between these sorted proteins also remain to be explored in different patho-physiological conditions.

## Supporting Information

Figure S1 **Conditioned media from myoblasts or myotubes were divided into two fractions.** One fraction was directly used for ELV extraction by ultracentrifugation. The remaining fraction was filtered through a 0.2 µm filter before ultracentrifugation. Then ELV size distribution of all fractions was measured by photon correlation spectroscopy using the Zetasizer NanoS (Malvern Instruments, UK) at 20°C. As indicated, the filtering step removed large particles above 300 nm.
(TIF)

Figure S2 **Comparison of C2C12 myoblast proliferation in normal DMEM 10% FBS or with DMEM depleted-exosome 10% FBS.** A_left, cell index determination with the xCellingence System; right microscopy-based images of C2C12 myoblasts at 80% confluence. B_ C2C12 myoblast size analysis after 48 h proliferation.
(TIF)

Figure S3 **Light microscopy-based images of undifferentiated myoblasts (day 0) and differentiating cells at various time points (days 2 (2d), 4 (4d), and 8 (8d)) following serum starvation and induction of the myogenic program ((2% Horse Serum (HS) containing exosomes or exosome-depleted).** Bar = 100 nm.
(TIF)

Figure S4 **Cell cycle analysis.** Myoblasts in suspension were fixed in ethanol 70% then treated with 10 µg/ml RNAse H (Promega, Charbonnières, France) in PBS 1X during 1H before propidium iodide (Sigma Aldrich) was added (50 µg/ml). Flow cytometry analysis of 5,000 cells was performed on a FACSCantoII flow cytometer (BD Biosciences) and data were recovered using the FACSDiva software v6.1.2 (BD Biosciences). DNA content was determined using FlowJo software v8.8.6 (http://www.flowjo.com).
(TIF)

Figure S5 **C2C12 myoblast size analysis A_C2C12 myoblast size quantification after 24** **h proliferation in 96-well plates, either with ELV-MB or ELV-MT, determined by using the Scepter 2.0 handheld automated cell counter from Millipore.** (see legend of Figure 5C). Cell sizes under 8 µm represent dead cells or aggregates. B_Representative light microscopy-based images of proliferating myoblasts 24 h post-incubation either with ELV-MB or ELV-MT, showing that ELVs treatment did not affect cell morphologies.
(TIF)

Figure S6 **C2C12 myoblasts were seeded in 12-well plates (2500 cells/cm^2^) and grown in DMEM (n = 6 replicates).** Twenty-four hours later, cells were grown in exosome-depleted DMEM and incubated with different concentrations of ELV-MB or ELV-MT (µg/ml of medium) for an additional 24 h. A_Cells were washed in PBS to remove dead cells and total RNA was extracted and quantified by using a NanoDrop (thermo Scientific). The quantity of total RNA is proportional to the cell number. As shown, the quantity of total RNA recovered from cells treated with ELV-MB did not significantly differ from the quantity of total RNA extracted from cells treated with ELV-MT (*p* value>0.5 from student t-test). B_Twenty-four hours after ELV treatments, each well was washed in PBS and cells were trypsinised. They were resuspended in 400 µl of DMEM. Aliquots of 40 µl were diluted with 40 µl trypan blue (0.4% in PBS). The viable cells were counted (n = 3 replicates). As shown, the total number of viable cells after ELV-MB treatments was not significantly different from the total number of viable cells after ELV-MT treatment (*p* value>0.5 from student t-test). Data from A and B are from independent experiments.
(TIF)

Figure S7 **Venn Diagrams showing the number of overlapping proteins between ELV-MB and ELV-MT.**
(TIF)

Table S1 **Differential analysis of ELV-MB and ELV-MT proteomes.**
(XLS)

Table S2 **The 71 proteins commonly identified both in this study and in the study of Guescini et al. 2010, in ELVs released from C2C12 myoblasts.**
(XLS)
