# Correction: Proteomic Analysis of C2C12 Myoblast and Myotube Exosome-Like Vesicles: A New Paradigm for Myoblast-Myotube Cross Talk?

**DOI:** 10.1371/annotation/ecd1e074-2618-4ad0-95c0-efdb467c714b

**Published:** 2014-01-29

**Authors:** Alexis Forterre, Audrey Jalabert, Emmanuelle Berger, Mathieu Baudet, Karim Chikh, Elisabeth Errazuriz, Joffrey De Larichaudy, Stéphanie Chanon, Michèle Weiss-Gayet, Anne-Marie Hesse, Michel Record, Alain Geloen, Etienne Lefai, Hubert Vidal, Yohann Couté, Sophie Rome

The graphs are missing from Supplementary Figures S3 and S5. Please see the correct version of the figures here:

Figure S3: 

Figure S5: 
