# Supplementary figures and images for: Proteomic Analysis of C2C12 Myoblast and Myotube Exosome-Like Vesicles: A New Paradigm for Myoblast-Myotube Cross Talk?
Source: PLoS One. 2014 Jan 2;9(1):e84153. doi: 10.1371/journal.pone.0084153 (PMC3879278; doi:10.1371/journal.pone.0084153)

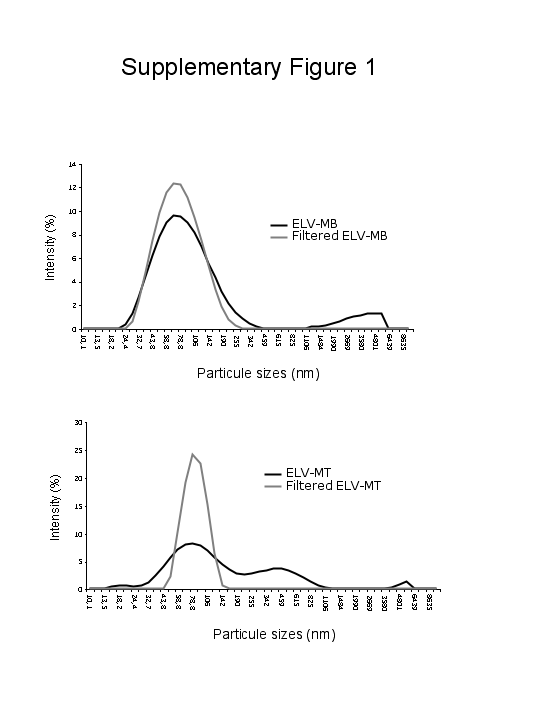

Supplement: Figure S1 — Conditioned media from myoblasts or myotubes were divided into two fractions. One fraction was directly used for ELV extraction by ultracentrifugation. The remaining fraction was filtered through a 0.2 µm filter before ultracentrifugation. Then ELV size distribution of all fractions was measured by photon correlation spectroscopy using the Zetasizer NanoS (Malvern Instruments, UK) at 20°C. As indicated, the filtering step removed large particles above 300 nm. (TIF) [file pone.0084153.s001.tif]

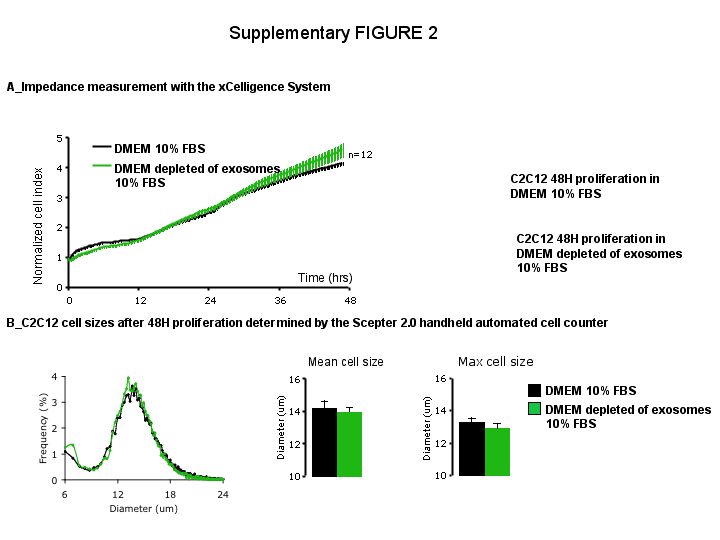

Supplement: Figure S2 — Comparison of C2C12 myoblast proliferation in normal DMEM 10% FBS or with DMEM depleted-exosome 10% FBS. A_left, cell index determination with the xCellingence System; right microscopy-based images of C2C12 myoblasts at 80% confluence. B_ C2C12 myoblast size analysis after 48 h proliferation. (TIF) [file pone.0084153.s002.tif]

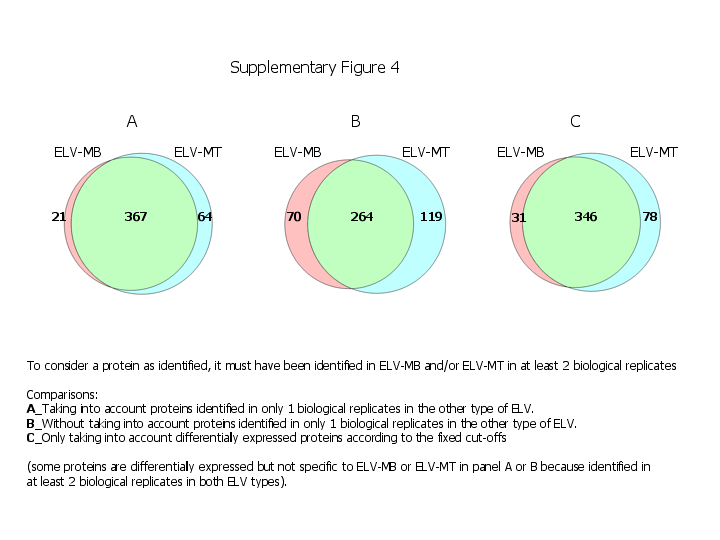

Supplement: Figure S4 — Cell cycle analysis. Myoblasts in suspension were fixed in ethanol 70% then treated with 10 µg/ml RNAse H (Promega, Charbonnières, France) in PBS 1X during 1H before propidium iodide (Sigma Aldrich) was added (50 µg/ml). Flow cytometry analysis of 5,000 cells was performed on a FACSCantoII flow cytometer (BD Biosciences) and data were recovered using the FACSDiva software v6.1.2 (BD Biosciences). DNA content was determined using FlowJo software v8.8.6 (http://www.flowjo.com). (TIF) [file pone.0084153.s004.tif]

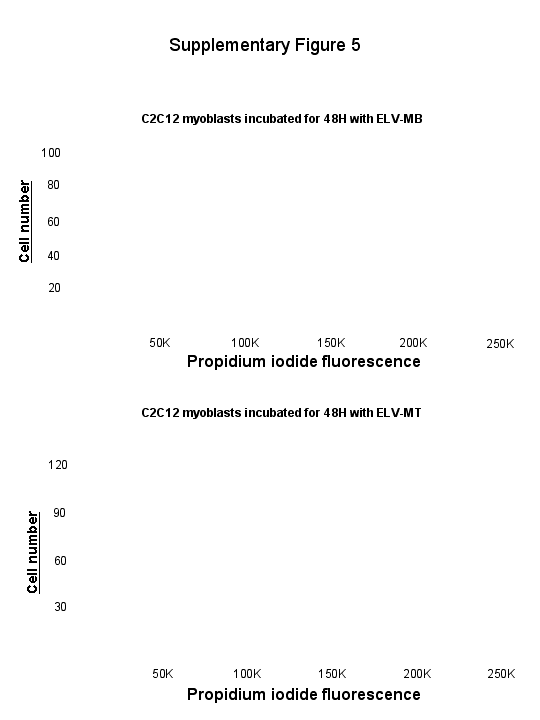

Supplement: Figure S5 — C2C12 myoblast size analysis A_C2C12 myoblast size quantification after 24 h proliferation in 96-well plates, either with ELV-MB or ELV-MT, determined by using the Scepter 2.0 handheld automated cell counter from Millipore. (see legend of Figure 5C). Cell sizes under 8 µm represent dead cells or aggregates. B_Representative light microscopy-based images of proliferating myoblasts 24 h post-incubation either with ELV-MB or ELV-MT, showing that ELVs treatment did not affect cell morphologies. (TIF) [file pone.0084153.s005.tif]

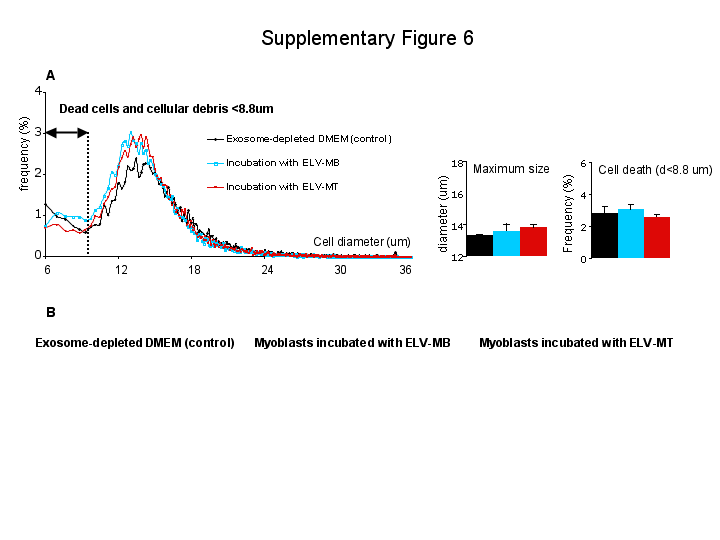

Supplement: Figure S6 — C2C12 myoblasts were seeded in 12-well plates (2500 cells/cm2) and grown in DMEM (n = 6 replicates). Twenty-four hours later, cells were grown in exosome-depleted DMEM and incubated with different concentrations of ELV-MB or ELV-MT (µg/ml of medium) for an additional 24 h. A_Cells were washed in PBS to remove dead cells and total RNA was extracted and quantified by using a NanoDrop (thermo Scientific). The quantity of total RNA is proportional to the cell number. As shown, the quantity of total RNA recovered from cells treated with ELV-MB did not significantly differ from the quantity of total RNA extracted from cells treated with ELV-MT (p value>0.5 from student t-test). B_Twenty-four hours after ELV treatments, each well was washed in PBS and cells were trypsinised. They were resuspended in 400 µl of DMEM. Aliquots of 40 µl were diluted with 40 µl trypan blue (0.4% in PBS). The viable cells were counted (n = 3 replicates). As shown, the total number of viable cells after ELV-MB treatments was not significantly different from the total number of viable cells after ELV-MT treatment (p value>0.5 from student t-test). Data from A and B are from independent experiments. (TIF) [file pone.0084153.s006.tif]

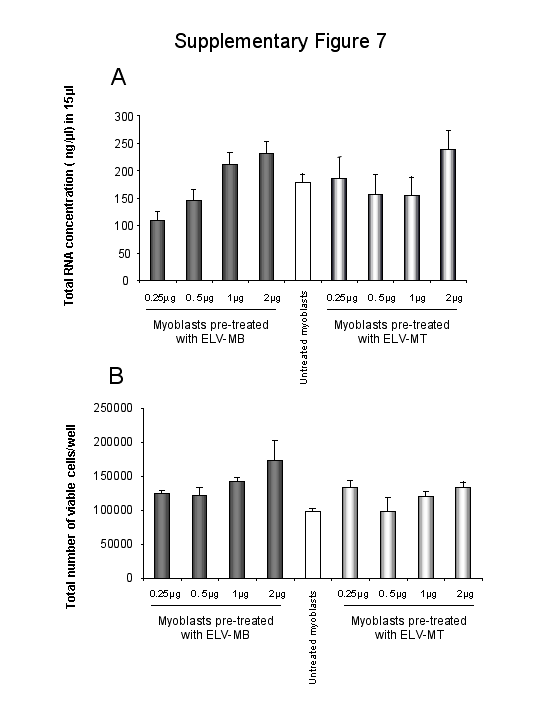

Supplement: Figure S7 — Venn Diagrams showing the number of overlapping proteins between ELV-MB and ELV-MT. (TIF) [file pone.0084153.s007.tif]
