# Supplementary figures and images for: Correction: Proteomic Analysis of C2C12 Myoblast and Myotube Exosome-Like Vesicles: A New Paradigm for Myoblast-Myotube Cross Talk?
Source: PLoS One. 2014 Jan 29;9(1):10.1371/annotation/ecd1e074-2618-4ad0-95c0-efdb467c714b. doi: 10.1371/annotation/ecd1e074-2618-4ad0-95c0-efdb467c714b (PMC3906386; doi:10.1371/annotation/ecd1e074-2618-4ad0-95c0-efdb467c714b)

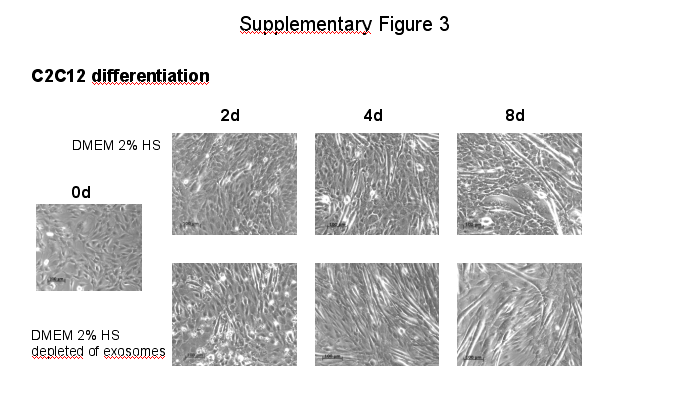

Supplement: Supplementary file 1 [file pone.ecd1e074-2618-4ad0-95c0-efdb467c714b.s001.tif]

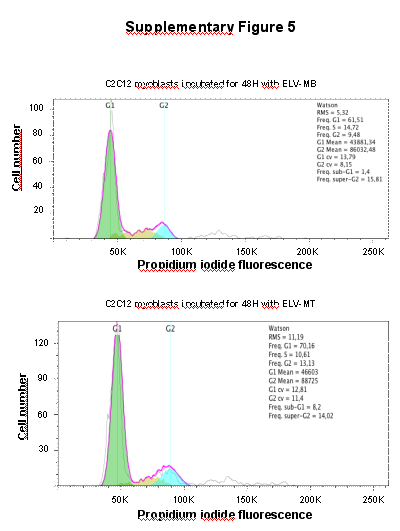

Supplement: Supplementary file 2 [file pone.ecd1e074-2618-4ad0-95c0-efdb467c714b.s002.tif]
